# Digital Assessment and Classification of Wine Faults Using a Low-Cost Electronic Nose, Near-Infrared Spectroscopy and Machine Learning Modelling

**DOI:** 10.3390/s22062303

**Published:** 2022-03-16

**Authors:** Claudia Gonzalez Viejo, Sigfredo Fuentes

**Affiliations:** Digital Agriculture, Food and Wine Group, School of Agriculture and Food, Faculty of Veterinary and Agricultural Sciences, University of Melbourne, Melbourne, VIC 3010, Australia; sfuentes@unimelb.edu.au

**Keywords:** off-aromas, rapid methods, machine learning, wine quality

## Abstract

The winemaking industry can benefit greatly by implementing digital technologies to avoid guesswork and the development of off-flavors and aromas in the final wines. This research presents results on the implementation of near-infrared spectroscopy (NIR) and a low-cost electronic nose (e-nose) coupled with machine learning to detect and assess wine faults. For this purpose, red and white base wines were used, and treatments consisted of spiked samples with 12 faults that are traditionally formed in wines. Results showed high accuracy in the classification models using NIR and e-nose for red wines (94–96%; 92–97%, respectively) and white wines (96–97%; 90–97%, respectively). Implementing new and emerging digital technologies could be a turning point for the winemaking industry to become more predictive in terms of decision-making and maintaining and increasing wine quality traits in a changing and challenging climate.

## 1. Introduction

Wine quality traits are characterized by several factors, such as physicochemical parameters, sensory descriptors, and aroma profiles, which can present faults (off-flavors/off-aromas) due to various factors within the viticultural production, winemaking and bottling processes.

Table 1 shows some of the most common faults found in wine, along with their sensory detection threshold and spoilage concentration. These faults were reported as most common when using traditional winemaking techniques [1]. Although most of these compounds may be present in wines, they must be kept below the detectable/spoilage concentration to avoid lowering the wine quality perception by negatively affecting its sensory attributes. The presence of faults outside the limits may lead to not complying with specific regulations and/or decreasing consumers’ acceptability, which has a negative economic impact on the wine industry [1,2].

Typically, the assessment of faults relies on the use of expensive equipment, such as gas chromatography-mass spectroscopy (GC-MS), which is time-consuming. Another approach to detecting faults is through the winemaker and/or a wine “experts” group. However, this tends to be subjective and is unable to quantify the specific concentration of each fault. Furthermore, this is carried out once the final product is ready, making it too late to make the most effective corrective actions to offer a high-quality wine. Recently, novel digital methods for food and beverages assessment have been developed using technologies such as sensor arrays (electronic noses and tongues) [3,4,5,6,7], computer vision, machine learning, and artificial intelligence. Some authors have used a combination of a commercial electronic tongue and sensory flash profiling to detect red wine faults derived by different microorganisms over time [8]. Furthermore, Macias et al. [9] used a commercial electronic nose (e-nose) with machine learning to detect acetic acid but used synthetic wine. Previously, it was shown that by implementing digital technologies, such as near-infrared spectroscopy (NIR), low-cost electronic noses (e-nose), and machine learning, it is possible to detect smoke contamination in canopies, berries [10], and smoke taint in wines due to bushfire events [11,12]. The latter models presented accuracies higher than 90% for classification and with correlation coefficients of R > 0.95 for regression models to predict specific smoke-related compounds in berries and wines. Implementing these digital technologies was the first attempt to transform the grape and wine industry production from traditionally reactive to a more predictive process using smart decision making [13,14]. For fault detection in fermented beverages, previous research has shown high accuracy for artificial intelligence (AI) tools using NIR, e-noses, and machine learning for beer [15]. Specifically, Gonzalez Viejo et al. [15] developed a method to detect 18 different faults in beer using a low-cost e-nose coupled with artificial neural networks; this technique is able to predict the level of concentration of faults and the specific compounds.

This study aimed to develop novel digital methods to automatically detect red and white wine faults using a near-infrared spectroscopy device and a portable and low-cost e-nose coupled with machine learning modeling.

**Table 1:** List of common faults found in wine, their associated aroma, contamination stage within the production line and concentration information. NR: Not reported.

Compound	Common Name	Associated Aroma	Contamination Stage/Source	Detection Threshold (mg L^−1^)	Spoilage Concentration (mg L^−1^)	Reference
2,6-Dichlorophenol	2,6-DCP	Chlorine/Plastic	Manufacturing and storage	Odor: 3.2 × 10^−5^ Flavor 4.8 × 10^−5^	NR	[16]
6-Chloro-o-cresol	6 CoC	Chlorine/Plastic	Manufacturing and storage	Odor: 7.4 × 10^−5^	NR	[17]
4-Ethylcatechol	4-EC	Leather/Horse/Phenolic	Brettanomyces contamination	Odor: 0.06–0.82	NR	[18]
4-Ethylphenol	4-EP	Leather/Stable/Horse	Brettanomyces contamination	Odor: 0.22–1.2 Flavor: 4	>0.43	[19,20]
4-Ethylphenol, 4-Ethylguaiacol	Brettanomyces mix	Medicinal/Smokey/Spicy	Brettanomyces contamination	Odor: 0.32	NR	
Guaiacol	Guaiacol	Smokey	Smoke taint (field)	Odor: 9.5 × 10^−3^–7.5 × 10^−2^	>0.08	[21]
2,4,6 Trichloroanisole	TCA	Must taint/ Moldy	Ageing Storage (cork)	Odor: 3 × 10^−3^–1.5 × 10^−2^ Flavor: 1.2 × 10^−2^	NR	[19,22]
Acetaldehyde	Acetaldehyde	Green apple/Bread/ Grass	Microbial contamination Poor yeast health Oxidation	Odor: 40–100 Flavor: 100–125	>125	[23,24,25,26]
Acetic acid	Acetic acid	Sour/Vinegar/ Tangy/Pungent	Spoilage bacteria Wild yeast	600–900	400–1500	[17,27,28]
Ethyl acetate	Ethyl acetate	Fruity/Ethereal/Weed/Green	Microbial Contamination	Odor: 10 Flavor: 160	>150	[17,19]
Dimethyl disulfide	DMDS	Onion/Cabbage/Sulfur	Fermentation	Odor: 0.02–0.05	NR	[27,29,30]
Methyl mercaptan	Methanethiol	Garlic/Cabbage/Egg	Fermentation Autolysis; Poor yeast health	Odor: 2 × 10^−5^	NR	[17,27]

## 2. Materials and Methods

### 2.1. Samples Description

A dry red (pH = 3.6; alcohol: 11.5%; Coolabah, Milsons Point, NSW, Australia) and fresh dry white wine (pH = 3.3; alcohol: 9.5%; Sonata Estate; Endeavour Group, Surry Hills, NSW, Australia) were used as base wines to spike with 12 different common faults in wine (samples were spiked with one fault each), which were used as treatments. Additionally, 24 replicates of control samples with no faults added were used for each wine as follows: two casks of wine were used, 12 control replicates were obtained from each cask (two at different times from opening and every 1 h). Wine samples were spiked using three concentration levels labeled as low, medium and high. However, due to similarities in medium and high concentrations for chemical fingerprinting and voltage of volatile compounds given by the saturation thresholds above the medium level, values from these two concentrations (medium-high) were averaged for analysis and modeling purposes. Concentrations were calculated based on volume; the acquired compound vials were prepared as 3 mL solutions; therefore, low concentration samples were prepared with 1 mL, medium concentration with 2 mL and high concentration with 3 mL. Specific concentrations used for each compound and level are shown in Table 2.

### 2.2. Near-Infrared Measurements

**Figure 1:**
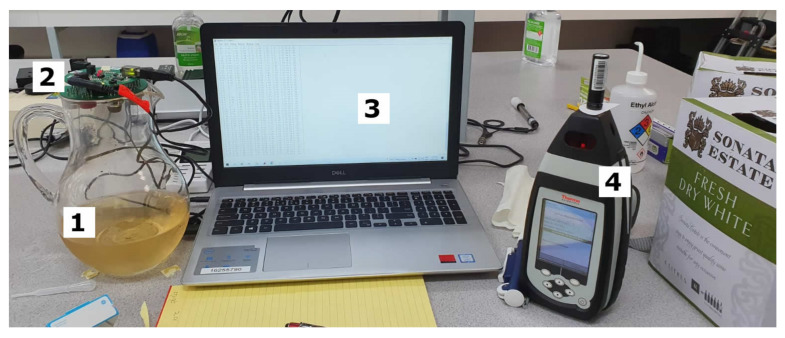
Example of setup used to measure: (**1**) wine samples using a (**2**) low-cost electronic nose connected to a (**3**) personal computer to collect and save data, and (**4**) MicroPHAZIR™ RX analyzer.

A MicroPHAZIR™ RX analyzer (Thermo Fisher Scientific, Waltham, MA, USA) was used to obtain the chemical fingerprinting (Figure 1) of a total of 24 replicates for each wine (red and white) of the control samples were measured in triplicates (N = 72), while three measurements were conducted in three replicates per concentration per spiked sample (n = 9 per concentration; N = 27 per fault). The absorbance values were measured within the 1596–2396 nm range following the method described by Gonzalez Viejo et al. [31] for all samples using a filter paper Whatman^®^ (Whatman plc. Maidstone, UK) qualitative grade 1 and 55 mm diameter; the filter paper was measured dry and then submerged in the sample; the absorbance values of the dry filter were subtracted from those of the wet filter to obtain only the wine readings. The curves from mean values of each treatment using raw data were plotted using Matlab^®^ R2021a (Mathworks, Inc., Natick, MA, USA).

### 2.3. Electronic Nose Measurements

As shown in Figure 1, a low-cost e-nose composed of an array of nine different gas sensors, such as (i) MQ3: alcohol, (i) MQ4: methane (iii) MQ7: carbon monoxide, (iv) MQ8: hydrogen, (v) MQ135: ammonia/alcohol/benzene, (vi) MQ136: hydrogen sulfide, (vii) MQ137: ammonia, (viii) MQ138: benzene/alcohol/ammonia, and (ix) MG811: carbon dioxide was used to measure wine samples. A total of 24 replicates were measured for control samples and three replicates per concentration for each fault. The e-nose is able to capture the voltage of volatile compounds detected by each sensor every 0.5 s. The output data were analyzed using a code written in Matlab^®^ R2021a by the Digital Agriculture Food and Wine group from The University of Melbourne (DAWF-UoM). As described by Gonzalez Viejo et al. [32], the code is able to display the curves of each sensor and sample, from which the stable segment of the highest peak is selected. The code divides this segment into 10 subsections and obtains the mean of each, providing 10 mean values per sensor and per sample.

### 2.4. Statistical Analysis and Machine Learning Modelling

The e-nose data were analyzed using ANOVA to assess significant differences between samples using Tukey’s honest significant difference (HSD) as post-hoc test (α = 0.05). Means and standard error (SE) were plotted as stacked bars with letters of significance to ease visualization. Means of low concentration and medium-high were plotted separately to compare each type of wine (red and white).

**Figure 2:**
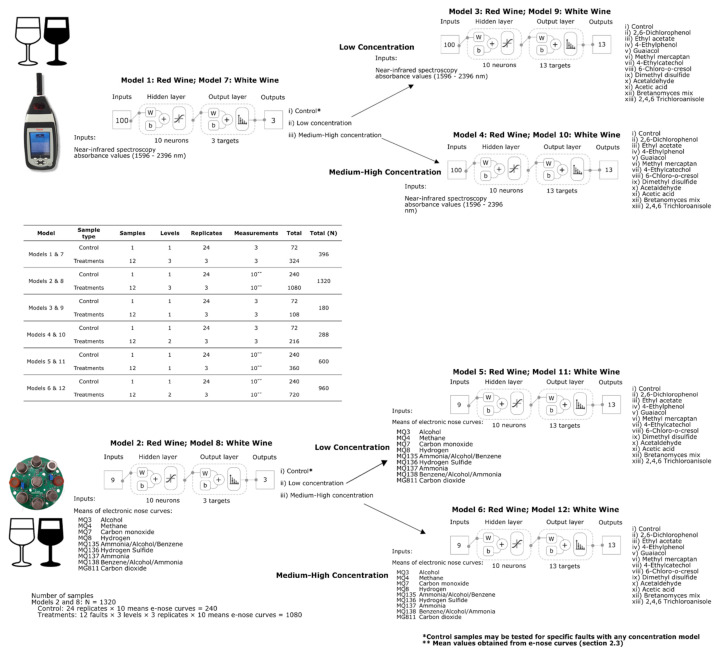
Diagrams of the digital instrumentation measurements and two-layer feedforward models with a tan–sigmoid function in the hidden layer and a Softmax function in the output layer for Models 1 (low concentration) and 2 (high concentration), and Models 3 (low concentration) and 4 (high concentration). Abbreviations: W: weights; b: bias.

Six classification ML models for each type of wine were developed using artificial neural networks. As described in previous publications from Gonzalez Viejo et al. [15,33], a customized Matlab^®^ R2021a code developed by the DAFW-UoM group was used to train the models using 17 supervised algorithms classified as two algorithms based on backpropagation with Jacobian derivatives, 11 based on backpropagation with gradient derivatives and four using weight/bias training functions. The best models were selected based on the highest accuracy and performance calculated using means squared error (MSE) to assess the absence of overfitting. All models were developed using Bayesian Regularization algorithm, which resulted in the best models. Models 1 and 7 for red and white wines, respectively, were developed using raw NIR absorbance values (1596–2396 nm) as inputs to classify samples into (i) control (no faults), (ii) low, and (iii) medium–high concentration of faults. Samples were divided using interleaved indices into training (70%; N = 277) and testing (30%; N = 119). Likewise, Models 2 and 8 for red and white wines, respectively, were developed to classify samples into (i) control (no faults), (ii) low, and (iii) medium–high concentration of faults but using e-nose outputs as inputs (Figure 2). Samples were also divided using interleaved indices into training (70%; N = 924) and testing (30%; N = 396).

Once the concentration of faults is predicted using either of the previously described models (Models 1, 2, 7 and 8), Models 3, 5 for red wine, 9 and 11 for white wines should be used to identify the specific fault present in the low concentration samples. This is achieved using NIR absorbance values (1596–2396 nm) as inputs for Models 3 and 9 and e-nose outputs for Models 5 and 11. Likewise, Models 4, 6 for red wine, 10 and 11 for white wines should be used to identify the specific fault present in the high concentration samples using NIR absorbance values (1596–2396 nm) as inputs for Models 4 and 6 and e-nose outputs for Models 10 and 12 (Figure 2). For all the models, samples were also divided using interleaved indices into training (70%) and testing (30%); the specific number of samples for these models will be presented in Results and Discussion section. For all models, 10 neurons resulted in the best accuracies and performance after a neuron trimming exercise testing three, five, seven, and 10 neurons.

## 3. Results and Discussion

**Figure 3:**
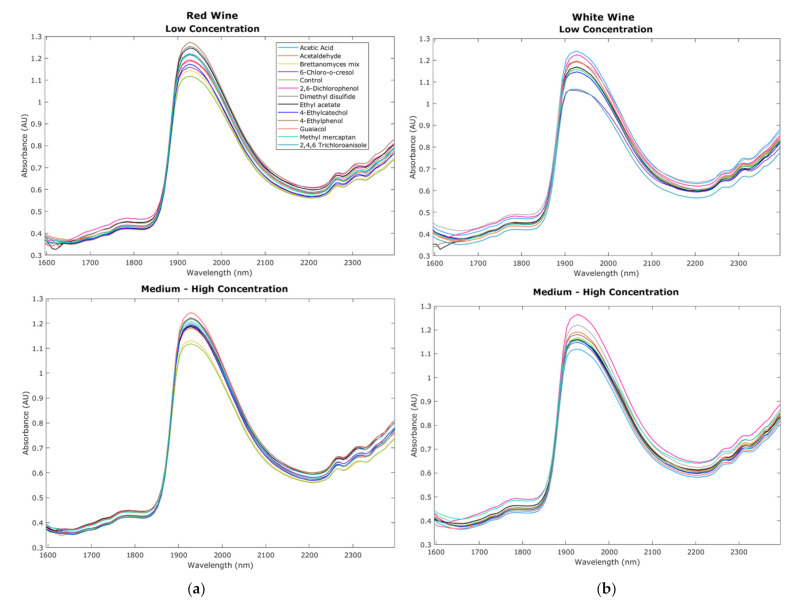
Chemical fingerprinting obtained from the near-infrared spectroscopy measurements within the 1596–2396 nm range. Curves display the results from (**a**) red wine and (**b**) white wine raw absorbance values from low and medium–high concentration samples.

Figure 3a,b show the NIR curves from the red and white wine samples with overtones such as carboxylic acids (1905 nm), water (1795 nm, 1807 nm and 1940–1945 nm), alcohols (1807 nm), ammonia, glucose, polysaccharides, and proteins carboxylic acids (>2200 nm) [34,35]. All these compounds are commonly found in wine; furthermore, overtones for aromatic hydrocarbons (1775 nm) and amines (1968 and 1982 nm) were also found in the wine samples studied [34]. It can be observed that the red and white control wines (green line), despite having similar overtones, had different absorbance levels due to the variation in grape variety composition of berries pulp and skin, as well as compounds developed during fermentation and winemaking. Comparing low and medium–high concentrations of faults, for red wines, the absorbance of the different faults appears more variable for low concentrations than the medium–high concentrations in which most samples with faults had similar absorbance values based on the curves shown (Figure 3a). On the other hand, for white wine, both low and medium–high concentration samples showed variability in absorbance values (Figure 3b). Even though these curves represent only the mean values of all replicates for visual representation, which reduces variability among treatments, it can still be observed that the absorbance values of samples with faults for both red and white wines varied at different fault concentrations. Variability in the NIR measurements within the replicates may be due to different factors such as environmental effects when the wine is exposed, temperature variations, volatile compounds, and type of fault used, among others. On the other hand, variability within different treatments is due to different compounds and changes within the wine generated by the specific faults.

**Figure 4:**
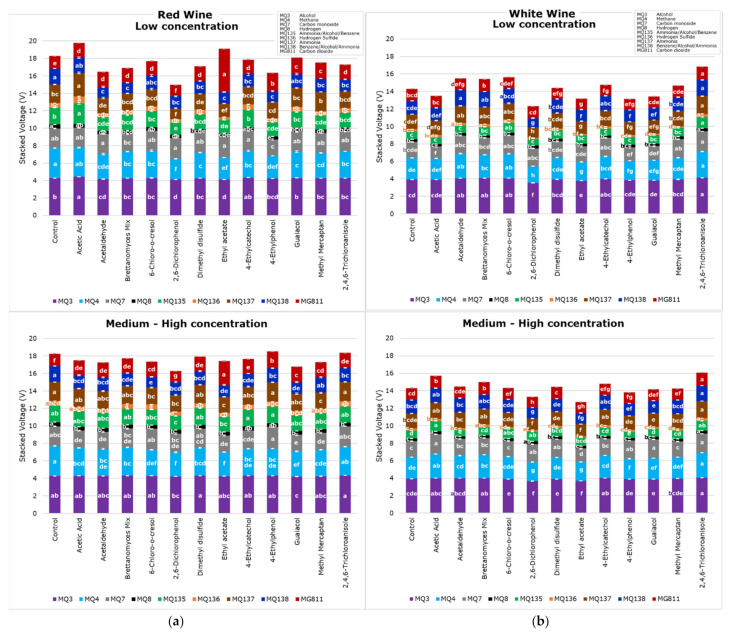
Electronic nose mean values from each gas sensor for (**a**) red and (**b**) white wine for low and medium–high concentration samples, showing the letters of significance (a–h) between samples based on Tukey’s honest significant difference (HSD) test (*p* < 0.05).

Figure 4 shows that the e-nose was able to find significant differences between samples for the nine sensors in both the red (Figure 4a) and white (Figure 4b) wines. Furthermore, it shows different results for low and medium–high concentration samples. It can be observed that the MQ3 sensor, which is sensitive to alcohol, had the highest voltage values, followed by MQ4, mainly sensitive to methane but also alcohol and smoke, compared to other sensors. While MQ8, which is sensitive to hydrogen, presented the lowest voltage values for all samples, followed by MQ136 (hydrogen sulfide). It is important to note that values for MG811 are inverse; therefore, the highest values mean the lowest carbon dioxide. It can also be observed that the red wine samples had higher voltage values than white wine for most gas sensors, which may be due to the more intense aroma compounds in the red wine sample as they had higher ethanol concentrations than the white wine. It was reported that ethanol in wine has an effect on the intensity of aroma compounds [36]. The higher voltage for the ammonia-sensitive sensors (MQ135 and MQ137) for the red wine compared to the white wine may be due to differences in their fermentation processes. Furthermore, there are visible differences between the voltage of low and medium–high concentrations of faults in both types of wines, which aids in the development of Model 1 to predict the level of concentration (low or medium–high) using the e-nose outputs as inputs.

**Figure 5:**
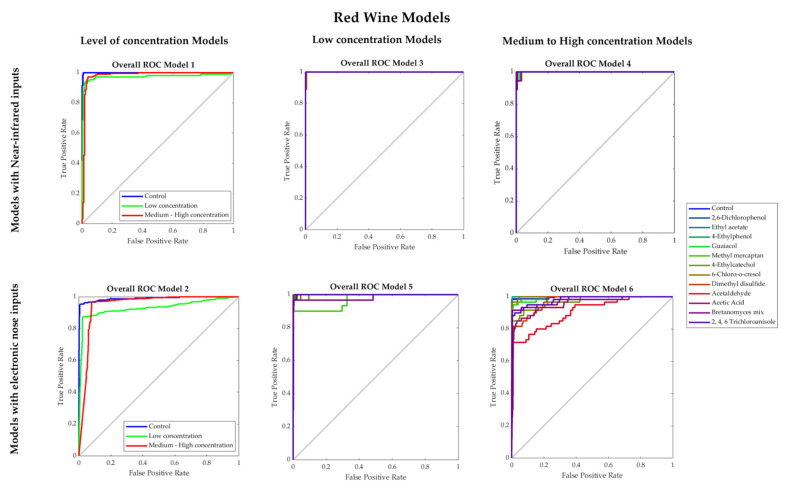
Receiver operating characteristics (ROC) curves of the overall Models 1–6 developed for red wine.

Table 3 shows that Models 1 and 2 had high overall accuracies (Model 1: 96%; Model 2: 94%) to classify red wine samples into control, low and medium–high concentrations of faults using NIR and e-nose inputs, respectively. Both models had high performance and no signs of overfitting as the training MSE values (Model 1: MSE < 0.001; Model 2: MSE = 0.02) were lower than the testing stage (Models 1–2: MSE = 0.08). Furthermore, in Figure 5, which shows the receiver operating characteristics (ROC) curves, it can be observed that for both Models 1 and 2, low concentration presented the lowest true-positive rate (Model 1: 0.93; Model 2: 0.87).

Models 3 and 4 developed using NIR inputs to predict the specific faults present in red wines for low (Model 3) and medium-high concentration (Model 4) had high overall accuracies (94% and 97%, respectively; Table 3). These models also showed no signs of overfitting based on the performance values. In Figure 5, it can be observed that in Model 3, the lowest true-positive rates were found for the control (0.88) and dimethyl disulfide (0.89), while in Model 4, 6-chloro-o-cresol and acetic acid had the lowest true-positive rate (0.89). On the other hand, Models 5 and 6 to predict faults in low (Model 5) and medium-high concentration (Model 6) using e-nose inputs also had high overall accuracies (97% and 92%, respectively) with no signs of overfitting according to the performance values. In the ROC curves (Figure 5), it can be observed that for Model 5, methyl mercaptan had the lowest true-positive rate (0.83), while in Model 6, the lowest rate was for acetaldehyde (0.72) and dimethyl disulfide (0.78), and acetic acid (0.80). This may be due to the oversaturation of these faults’ volatile compounds captured by the e-nose due to their high concentration levels.

These results show that both the chemical fingerprinting within the 1596–2396 nm range and volatile compounds measured with e-nose are effective to detect faults in red wine.

**Figure 6:**
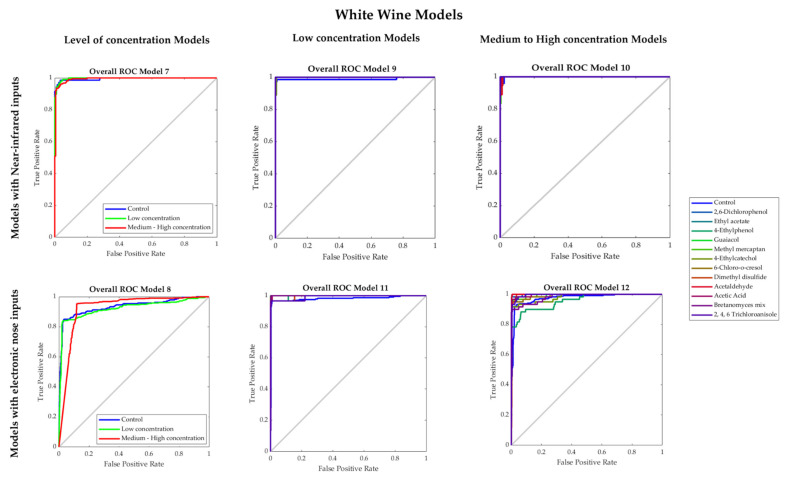
Receiver operating characteristics (ROC) curves of the overall Models 7–12 developed for white wine.

Table 4 shows the results from the machine learning models developed for white wine. Models 7 and 8 presented 95% and 90% overall accuracies, respectively, to predict the presence and concentration of faults using NIR (Model 7) and e-nose (Model 8) inputs. None of these two models presented any signs of overfitting as the training MSE values (Model 9: < 0.01; Model 10: 0.04) were lower than those from the testing stage (Model 9: < 0.04; Model 10: 0.12). Figure 6 shows the ROC curves of the models, it can be observed that the lowest true-positive rate in Model 7 was 0.94 for control and low concentration samples, while for Model 8, the control had the lowest true-positive rate (0.83).

Models 9 and 10, which were developed using the NIR inputs to predict the specific faults present in the white wine samples with low and medium–high concentration, respectively, had very high accuracies (Model 9: 97%; Model 10: 96%), with no signs of overfitting based on their performance values. According to the ROC curves (Figure 6), in Model 9, the lowest true-positive rates were from in 2, 6-dicholophenol (0.89), 4-ethylcatechol (0.89), and 2,4,6 tricholoanisole (0.89), while in Model 10, the lowest values were from methyl mercaptan (0.83), 6-cholo-o-cresol (0.90), and acetaldehyde (0.90). On the other hand, Models 11 and 12, developed using the e-nose inputs to predict the specific faults present in the white wine samples with low (Model 11) and medium–high concentration (Model 12), presented accuracies of 97% and 92%, respectively. Both models had high performance and no signs of overfitting. From the ROC curves, it was found that 4-ethylcatechol (0.93), acetaldehyde (0.93), and 2,4,6 tricholoanisole (0.93) had the lowest true-positive rates in Model 11. Moreover, in Model 12, the lowest true-positive rates were given by the 4-ethylphenol (0.78) and Brettanomyces mix (0.88).

The overall accuracies obtained in these models (> 90%) were similar to those reported for other fermented beverages using an e-nose, such as to predict alcohol content in beer (95%) [37], faults in beer (> 95%) [15], aroma intensity in beer (R > 0.93) [38], aroma intensity in wine (R > 0.97), smoke-derived compounds in wine (R = 0.98), roasting intensity (98%), and aroma intensity (R = 0.99) in coffee [32].

The models were developed using samples with a single fault because it is unusual to find more than one contamination source that may produce several faults in the same product. If this were the case, it would indicate a serious issue in the vineyard and/or winery that leads to the production of very low-quality wines. However, the ANN models developed, being multi-category, allow identifying more than one fault within the samples as they provide values within a 0–1 scale indicating the proportion of each identified fault. In that case, the importance of the models is on the sensitivity of the detection at levels that are even below common sensory perception.

The models were developed with cask wine as a base wine, which is typically a blend. Further research may be conducted to assess the accuracy with specific wine varieties and vintages. However, previous research on beer has shown that AI models can successfully differentiate the type of fermentation using other beers different from those used to develop the original model [39]. Additionally, the models developed in this study may be fed with data from other varieties and vintages to further generalize the models.

The high accuracy of the models showed that an outlier analysis was not necessary, which confirms that the proposed methods (NIR and e-nose) are reliable, reproducible, and consistent. The accuracies of all models for red and white wines developed using NIR, and e-nose inputs were similar, showing that both devices are suitable for detecting wine faults. However, the proposed e-nose device is more affordable and may be installed at any stage of the production line for the early detection of faults. This would allow the development of machine learning models to create digital twins to integrate and automate an assessment system as a low-cost, reliable, and convenient method to assess all batches produced to detect and solve any faults within each stage of the process.

## 4. Conclusions

The use of digital technologies in the winemaking industry could be a turning point for accurate and real-time assessment of the fermentation processes to detect the compositional changes of wine objectively during winemaking. The use of NIR and e-noses have the advantage of transforming the winemaking industry from being reactive to more predictive, avoiding the formation of off-flavors and aromas using artificial intelligence (AI) from the maceration of the grapes through the fermentation process to bottling. One important advantage of these AI models is the potential creation of digital twins based on grape production at the vineyard stage and through the harvest and vinification processes. With the deployment of these digital twins, it could be possible to run season simulations based on predicted weather data and water availability to optimize grape quality traits and minimize the formation of off-flavors and aromas at different stages of grape production, fermentation, and during the winemaking process.

## Figures and Tables

**Table 2:** Concentrations used for each treatment and faults to spike red and white wines.

Compound	Low Concentration in 750 mL of Wine * (mg L^−1^)	Medium Concentration in 750 mL of Wine * (mg L^−1^)	High Concentrationin 750 mL of Wine * (mg L^−1^)
2,6-Dichlorophenol	1.33 × 10^−4^	2.67 × 10^−4^	4.00 × 10^−4^
6-Chloro-o-cresol	1.33 × 10^−4^	2.67 × 10^−4^	4.00 × 10^−4^
4-Ethylcatechol	1.33	2.67	4.00
4-Ethylphenol	0.33	0.67	1.00
Brettanomyces mix (4-Ethylphenol, 4-Ethylguaiacol)	0.28	0.55	0.83
Guaiacol	0.04	0.08	0.12
2,4,6 Trichloroanisole	2.33 × 10^−6^	4.67 × 10^−6^	7 × 10^−6^
Acetaldehyde	40	80	120
Acetic acid	330	670	1000
Ethyl acetate	50	100	150
Dimethyl disulfide	0.18	0.36	0.54
Methyl mercaptan	3.33 × 10^−3^	6.67 × 10^−3^	0.01

* Compounds were mixed in 750 mL of wine to make the equivalent concentration in mg L^−1^.

**Table 3:** Accuracy and performance based on means squared error (MSE) of machine learning models 1–6 developed for red wine.

Stage	Samples	Accuracy	Error	Performance (MSE)
Model 1: Red Wine Concentration level—NIR inputs				
Training	277	100%	0.0%	<0.001
Testing	119	87.4%	12.6%	0.08
Overall	396	96.2%	3.8%	-
Model 2: Red Wine Concentration level—Electronic nose inputs				
Training	924	96.8%	3.2%	0.02
Testing	396	86.9%	13.1%	0.08
Overall	1320	93.8%	6.2%	-
Model 3: Red Wine Low concentration faults—NIR inputs				
Training	126	94.4%	5.6%	<0.001
Testing	54	94.4%	5.6%	<0.01
Overall	180	94.4%	5.6%	-
Model 4: Red Wine Medium-high concentration faults—NIR inputs				
Training	202	100%	0.0%	<0.001
Testing	86	88.5%	11.5%	0.01
Overall	288	96.5%	3.5%	-
Model 5: Red Wine Low concentration faults—Electronic nose inputs				
Training	420	99.5%	0.5%	<0.001
Testing	180	92.2%	7.8%	0.01
Overall	600	97.3%	2.7%	-
Model 6: Red Wine Medium-high concentration faults—Electronic nose inputs				
Training	672	96.0%	4.0%	<0.001
Testing	288	82.6%	17.4%	0.02
Overall	960	92.0%	8.0%	-

**Table 4:** Accuracy and performance based on means squared error (MSE) of machine learning models 1–6 developed for white wine.

Stage	Samples	Accuracy	Error	Performance (MSE)
Model 7: White Wine Concentration level—NIR inputs				
Training	277	100%	0.0%	<0.001
Testing	119	84.9%	15.1%	0.08
Overall	396	95.5%	4.5%	-
Model 8: White Wine Concentration level—Electronic nose inputs				
Training	924	93.6%	6.4%	0.04
Testing	396	81.6%	18.4%	0.12
Overall	1320	90.0%	10.0%	-
Model 9: White Wine Low concentration faults—NIR inputs				
Training	126	100%	0.0%	<0.001
Testing	54	90.7%	9.3%	<0.01
Overall	180	97.2%	2.8%	-
Model 10: White Wine Medium-high concentration faults—NIR inputs				
Training	202	100%	0.0%	<0.001
Testing	86	87.4%	12.6%	0.02
Overall	288	96.2%	3.8%	-
Model 11: White Wine Low concentration faults—Electronic nose inputs				
Training	420	99.5%	0.5%	<0.001
Testing	180	90.0%	10.0%	0.01
Overall	600	96.7%	3.3%	-
Model 12: White Wine Medium-high concentration faults—Electronic nose inputs				
Training	672	96.7%	3.3%	<0.01
Testing	288	81.2%	18.8%	0.03
Overall	960	92.1%	7.9%	-

## Data Availability

Data and intellectual property belong to The University of Melbourne; any sharing needs to be evaluated and approved by the University.
